# Protocol to evaluate the effectiveness of the implementation of transdiagnostic cognitive behavioural therapy for emotional disorders in primary care and its mechanisms of change: a randomized step-wedge clinical trial (PsicAP-CV)

**DOI:** 10.1371/journal.pone.0320857

**Published:** 2025-04-17

**Authors:** Roger Muñoz-Navarro, Virtudes Pérez-Jover, Gabriel Esteller-Collado, Carlos Van-der Hofstadt Román, Monika Salgueiro, Anna Llorca-Mestre, Elisabeth Malonda-Vidal, Vera Canet-Cortell, M. José Moraga-García, Ainhoa Coloma-Carmona, María Carpallo-González, Maider Prieto-Vila, Sara Barrio-Martínez, Ángel Aguilera-Martín, Mario Gálvez-Lara, Francisco Jurado-González, Elisa Aguirre, César González-Blanch, Paloma Ruíz-Rodríguez, Juan Antonio Moriana, Paula Samper-García, María Vicenta Mestre-Escrivá, Antonio Cano-Vindel

**Affiliations:** 1 Departamento de Personalidad, Evaluación y Tratamientos Psicológicos, Facultad de Psicología, Universidad de Valencia, Valencia, España; 2 Departamento de Psicología de la Salud, Universidad Miguel Hernández, Elche, España; 3 Instituto de Investigación Sanitaria y Biomédica de Alicante (ISABIAL), Alicante, Spain; 4 Unidad de Psicología Hospitalaria. Hospital General Universitario Dr. Balmis, Alicante, España; 5 Departamento de Psicología Clínica y de la Salud y Metodología de Investigación. Facultad de Psicología, Universidad del País Vasco UPV/EHU, Donostia-San Sebastián, España; 6 Departamento de Psicología Básica. Facultad de Psicología, Universidad de Valencia, Valencia, España; 7 Servicio de Salud Mental, Departamento de Salud Alicante-Hospital General, Alicante, España; 8 Facultad de Psicología, Universidad Complutense de Madrid, Madrid, España; 9 Departamento de Psicología, Universidad de Córdoba (España), Instituto Maimónides de Investigación Biomédica de Córdoba (IMIBIC), Hospital Universitario Reina Sofía, Córdoba, Spain; 10 Redbridge Talking Therapies Service-North East London NHS Foundation Trust, London, United Kingdom; 11 Centro de Salud Mental, Hospital Universitario Marqués de Valdecilla-IDIVAL, Santander, España; 12 Sector Embarcaciones, Centro de Atención Primaria, Servicio Madrileño de Salud, Tres Cantos, Madrid, España; University of Cologne: Universitat zu Koln, GERMANY

## Abstract

**Introduction:**

Emotional disorders (ED) are highly prevalent worldwide. The PsicAP trial, conducted in Spain, demonstrated the benefits of adding transdiagnostic cognitive behavioural therapy (TD-CBT) to treatment as usual (TAU) for the attention of these disorders in primary care (PC). Here we describe the design of a stepped wedge randomized controlled trial (RCT), inspired by the PsicAP project. This RCT has two main aims: 1) to test the implementation of the PsicAP protocol in a real clinical setting, further evaluating possible mechanisms of change underlying the efficacy of TD-CBT (emotional regulation, alliance, and therapist experience and training), and 2) to assess the impact of psychotropic medication use on neuropsychological function and treatment outcomes.

**Methods:**

A single-blind multicentre RCT with a stepped wedge design will be conducted. Participants (N=320) will be randomly assigned to an experimental group (EG1) or to a waiting list group (WG). The EG1 will receive immediate treatment and the WG will remain on the waiting list for 3 months. After this time, the WG will become a second experimental group (EG2) that will receive the same treatment as EG1 (PsicAP protocol). Patients will be assessed at post-treatment, at 3 and 9 months. Before starting treatment, a random subsample of patients (n=90) will undergo a neuropsychological assessment. These patients will be assigned to three groups based on their use of psychotropic medication at the time of randomization: no psychotropic medication, short-term use (< 3 months) and long-term use (≥ 3 months). All 90 participants will undergo the same neuropsychological assessment at one year. The RCT is expected to run from 01/05/23 to 01/10/25.

**Discussion:**

The results of this trial are expected to provide further support for the efficacy of the PsicAP TD-CBT protocol, as well as insight into the mechanisms of change that lead to the positive therapeutic outcomes of this protocol. In addition, this study will help determine the effects of short- and long-term psychotropic use on neuropsychological function and therapeutic outcomes. In short, it is hoped that this RCT will help to better understand how to implement evidence-based psychological treatment in the PC setting.

**Trial registration:**

EURADICT 2013-001955-11/ ISRCTN58437086.

## Introduction

Emotional disorders (EDs), such as depression or anxiety disorders, are the most prevalent mental health problems worldwide [1]. In Spain, the prevalence of these disorders is even higher than internationally [2]. Moreover, these disorders often manifest concurrently with each other or with other health conditions, indicating that comorbidity is a very common phenomenon [3]. EDs have a substantial impact on quality of life (QoL) and represent one of the leading causes of disability worldwide [4,5]. In addition, EDs entail significant economic costs to the healthcare system, both direct and indirect [6].

### Management of EDs in primary care

In most countries, EDs are usually addressed in the Primary Care (PC) setting by GPs [7,8], as this service is usually the gateway to the National Health System [9]. However, due to short consultation times, high care burden and poor training of GPs in psychological treatments and/or psychological diagnostic tools [9], it leads to approximately 40% of patients receiving no treatment at all and only 30% receiving evidence-based treatment [10].

Typically, EDs are addressed through what is known as treatment as usual (TAU), which usually consists of the pre-prescription of psychotropic medication and/or GP counselling. However, international guidelines for the treatment of these disorders is, in the first instance and for cases with mild to moderate severity, evidence-based psychological therapy, especially cognitive behavioural therapy (CBT) [11–13]. CBT has been shown to be a very effective and cost-effective alternative for the treatment of these disorders, achieving large effect sizes in both the short and long term [14,15]. In addition, some CBT approaches, such as Trandiagnostic CBT (TD-CBT), have also shown good efficacy [16] and a very interesting option for implementation in PC [17].

The first programme to implement this evidence-based psychological approach was the flagship “Improving Access to Psychological Therapies (IAPT)” project in the United Kingdom (UK), which has become an international benchmark for the treatment of these disorders. The project involves almost 11.000 therapists (both high and low intensity) providing CBT and other evidence-based psychological therapies in a stepped-care model in which specific treatment depends on the severity of symptoms [18]. A major advantage of the stepped-care approach is that it reduces overmedication and allows clinicians to select the most appropriate treatment for each case [18].

### The PsicAP clinical trial

The success of the IAPT programme encouraged the PsicAP Randomized Controlled Trial (RCT) in Spain, a project to evaluate the efficacy of evidence-based psychological therapy for the treatment of mild and moderate EDs in the Spanish PC setting [17]. This trial followed a double-arm, single-blind design, where participants (n=1061) were randomized to receive either TAU (control group) or TD-CBT+TAU (experimental group). The TD-CBT protocol consisted of 7 sessions of psychological therapy in a group format of 8–10 persons, delivered by a clinical psychologist (see information in the method section). The experimental treatment was more effective than TAU alone for reducing symptoms of depression, anxiety and somatizations, with medium to large effect sizes. The experimental treatment was also superior to TAU for improving QoL and functioning, with small to medium effect sizes. All these effects were maintained at 12 months, suggesting a long-lasting therapeutic effect. Furthermore, the observed rates of reliable recovery (≈50%) and impairment (≈3%) were similar to those achieved in the IAPT programme [10,18] and in a similar programme in Norway [19], highlighting the efficacy of the PsicAP protocol.

### Mechanisms of change of TD-CBT

In recent decades, transdiagnostic treatment approaches have received particular interest from the scientific community [16,19,20]. Transdiagnostic treatments for EDs typically include components that have been shown to be effective for anxiety disorders and depression, such as cognitive restructuring, behavioural activation or exposure [21,22]. A very recent meta-analysis has confirmed that TD-CBT is as effective as specific treatments in reducing anxiety and depression, both at post-treatment and at 6-month follow-up [16]. Moreover, TD-CBT can be administered in group format, obtaining a better cost-effectiveness ratio in the medium to long term compared to other traditional formats [23,24].

Due to the rise of evidence-based psychological therapies, there is a growing interest in understanding what are the mechanisms or processes of change in psychotherapy that drive positive therapeutic outcomes [25,26], as despite the fact that these treatments have shown good efficacy for the improvement of various mental disorders, there is still a significant number of patients who do not respond adequately to treatment [27,28]. Therefore, understanding the processes of change that underlie positive therapeutic outcomes may help to develop more effective psychological treatments. Thus, some studies have suggested that some of these components could be emotional regulation (ER) strategies, the therapeutic alliance, the therapist’s experience and some neuropsychological functions [29–34].

#### Emotion regulation strategies.

Emotion regulation strategies (ERS) have been shown to have a mediating role in EDs [29,30,35], which is why improved ER is an important target of TD-CBT [36,37]. According to some authors, CBT promotes improvement in certain cognitive and behavioural ERS, which in turn reduces symptoms [30,38]. Sloan et al. [37] suggested that the effectiveness of transdiagnostic therapies can be attributed to a reduction in certain maladaptive ERS (e.g., rumination, worry and suppression) and a simultaneous improvement in more adaptive strategies such as reappraisal and distraction. Other studies of transdiagnostic approaches have found that it is possible reducing comorbidities in people with anxiety disorders [39], an approach that has also proven effective when applied in a group format in PC [40].

A recent study based on data from the PsicAP trial evaluated the mechanisms of change that had the greatest impact on the efficacy of TD-CBT, showing the reduction in emotional symptoms and improvements in functioning and QoL were mainly attributable to a treatment-related decrease in maladaptive strategies such as worry, rumination, and negative metacognition [41]. Although that study found that adaptive ERS did not appear play a significant role in treatment outcomes, more data are needed to determine whether adaptive ERS play a mediating and moderating role in symptom reduction, QoL, and functioning.

#### Therapist experience and therapeutic alliance.

The therapeutic alliance has been shown to play a key role in treatment outcomes [34,42]. A meta-analysis examining the correlation between the therapeutic alliance and treatment outcomes found that the therapist’s contribution to the alliance appears to play an important role in improving patient outcomes [34]. Other studies have shown similar results [31,42]. In addition, some studies have pointed out that the therapeutic alliance is closely related to therapeutic dropout [43,44]. This is a key issue, as therapeutic dropout has been shown to be one of the main factors that reduce the effectiveness of psychological therapy [45–47].

Recent meta-analyses have shown that treatment discontinuation is a serious and widespread problem in psychotherapy, affecting approximately one in five patients (20%) [48,49]. Dropout rates are even higher in patients with anxiety and depressive symptoms [50]. In the PsicAP trial, approximately 60% of randomized patients completed the treatment protocol and/or attended the post-treatment assessment. In the experimental group, patients attended on average, five of the seven sessions. However, 11% of patients attended only 0–1 session. While the reasons for this are unclear, it clearly merits further investigation [17].

Therapist experience may also play an important role in treatment outcomes [51,52]. Our group has previously demonstrated the efficacy of highly trained clinical psychologists in the PsicAP trial [17]. However, in other projects such as IAPT, therapists have different levels of training (low and high intensity), showing that it is possible to safely and effectively include professionals from various health care backgrounds in mental health care, as long as they have received specialised training in evidence-based psychological treatment protocols [18].

With this in mind, in this trial we will assess the therapist-patient alliance, the alliance between patients within the group, and include therapists with different levels of training and experience. We hope that this will help to better understand positive therapeutic outcomes as well as factors leading to dropout.

#### Psychotropic medication use and neuropsychological alterations.

A recently published study on global trends in the consumption of psychotropic medication has pointed out that global sales of psychotropic drugs have increased at an average rate of 4% per year, from 28.5 daily doses per 1,000 inhabitants (DDH) in 2008 to 34.7 DDH in 2019 [53]. This has led to an overall increase in the prescription and consumption of psychotropic drugs, which in turn has led to an excessive overmedicalization of daily life [54,55]. In Spain, the latest report of the *Subdirectorate General for Health Information* has also confirmed this steady increase in the prescription of psychotropic medicines [2].

This high consumption of psychotropic drugs, as well as the resulting social, personal and economic costs, has led multiple authors to question the real efficacy of these drugs for the treatment of EDs [56–61], as although their use and prescription has increased, the overall trend of EDs has also increased [4,62]. A comprehensive meta-analysis comparing CBT against control conditions, other psychological therapies, pharmacotherapies and combination treatments for depression has recently been published [63]. Results showed that CBT and pharmacotherapy did not differ in the short term, but did differ in the long term (6 and 12 months follow-up), where CBT was more effective. Combined drug treatment was more effective than pharmacotherapies alone in the short and long term, but was not more effective than CBT alone at either time point. This has led to the suggestion that, although these medications may alleviate mood symptoms, they may also lead to emotional blunting that goes against the very essence of CBT [64]. Indeed, it is possible that withdrawal symptoms and some side effects such as increased anxiety, insomnia and agitation may make it difficult to engage in therapy [64–66]. In summary, the role of pharmacotherapy in the treatment of EDs is currently unclear.

Neuropsychological impairment is a common subjective complaint in EDs patients. Objective neuropsychological testing often reveals deficits in several cognitive domains, including processing speed, attentional processes, memory, verbal learning, executive function and working memory [67]. The presence of these impairments is considered a prognostic factor for poor response to treatment and reduced functional recovery [68]. Studies involving patients treated with psychotropics have consistently shown that these patients perform worse in verbal fluency, cognitive inhibition, visuospatial memory, verbal learning, working memory and executive function [32,69,70]. Some authors have reported an increased risk of cognitive impairment, including dementia, in patients treated with antidepressants for prolonged periods, especially selective serotonin reuptake inhibitors [71,72]. Tricyclic antidepressants, in addition to their effects on serotonin and noradrenaline reuptake, also have antihistamine effects that may produce drowsiness and some degree of sedation, which may negatively affect performance in activities of daily living, as evidenced by poorer performance on neuropsychological assessment tasks [73]. Prolonged treatment with tricyclic antidepressants has been shown to negatively affect cognitive performance [33,74], and these effects persist even after depressive and/or anxious symptoms have clinically improved [75]. Anticholinergic drugs, which are sometimes prescribed to treat depressive disorders, have been associated with poorer performance on neuropsychological assessment tasks, and exhibit greater cognitive impairment [76].

Many different cognitive processes are involved in the performance of CBT tasks, so medication-related cognitive impairment may negatively affect therapeutic outcomes. Although some studies have evaluated the effects of standard psychopharmacological treatments on neuropsychological processes, the results are still heterogeneous [61,77]. Despite the importance of this issue, to our knowledge no study has directly assessed the effects (especially the medium- to long-term effects) of antidepressants or anxiolytics on cognitive and psychosocial functioning in people with EDs.

### From PsicAP to PsicAP-CV: a stepped wedge ECA

The results of the PsicAP trial showed that it was possible to offer, from the PC setting, evidence-based psychological therapy in a safe and effective way to people with mild and moderate EDs [17]. However, this RCT did not analyze some variables that, as seen above, may be particularly relevant for therapeutic efficacy and dropout, such as the therapeutic alliance, the therapist’s experience and training, or the influence of the state of neuropsychological functions on the therapy process. Moreover, in the PsicAP trial, only a part of the included patients (experimental group) received the best available treatment (TD-CBT+TAU).

The PsicAP-CV trial aims to follow the path initiated by PsicAP, but adding some aspects that may help to complement and overcome the initial limitations. First, the PsicAP-CV trial will follow a Stepped Wedge design [78]. In this type of design, patients are assigned to an experimental group that receives immediate treatment (EG_1_) or to a waiting list group (WG) that, after a certain period of time, becomes a second experimental group (EG_2_) and receives the same treatment as the other group (see more details in the methods section). This allows all patients included in the RCT, either before or after, to receive the best available treatment, thus overcoming some important ethical issues. Furthermore, this design enables us to evaluate whether the effect of therapy varies based on the patient’s waiting time, as it is plausible that longer waiting periods may lead to more severe symptoms and greater impairment, thereby making recovery more challenging and complex [79,80]. This design also allows patients to be treated in a staggered manner, allowing more patients to receive treatment as time goes by, but without collapsing resources at a particular point in time. This is a fundamental aspect, as it is an effective RCT in the PC setting, where resources are very limited and there is often not enough staff or physical space to treat all patients simultaneously [9].

Another novelty of the PsicAP-CV trial is the addition of 3 extra sessions to the original PsicAP protocol (7 sessions). As previously noted, not all patients in the PsicAP trial achieved clinical recovery, so it is necessary to continue working to improve the therapeutic results. With this in mind, in the PsicAP-CV trial, only those people who have received the PsicAP therapy protocol (in group) and have not achieved clinical recovery will be offered three additional (individual) sessions on a monthly basis. It is expected that these sessions will help to work on certain specific contents that may contribute to the patient’s final recovery.

Finally, as mentioned above, the PsicAP-CV trial will measure the therapeutic alliance, include therapists with different levels of training and experience (similar to IAPT), and a sub-sample of patients will undergo a study of neuropsychological function (see more details in the methods section).

### Aims

#### General.

We aim to conduct an effectiveness RCT to assess the effectiveness of the PsicAP protocol in real-life clinical practice. This trial has two main objectives. First, to conduct a stepped wedge RCT to replicate the findings of the PsicAP trial, which showed that TD-CBT+TAU was more effective than TAU alone, in 6 PC centres in the Valencian Region (3 in Alicante and 3 in Valencia). Results were be assessed at three time points: immediately following treatment completion (posttreatment) and at 3 and 9 months (to determine whether the posttreatment results are maintained over time). The second main objective is to perform a neuropsychological assessment (working memory, executive function and attentional control) in a subsample of patients (n=90) included in the trial to determine how the short- and long-term use of psychotropic medications prior to treatment allocation affects cognitive function. A secondary objective is to evaluate potential mechanisms of change (ERS, therapist experience, and therapeutic alliance) that may influence the effectiveness of TD-CBT. The results of this study may help health care managers and clinicians to more precisely select the optimal treatment approach for specific patients or certain subpopulations, thus reducing the use of ineffective therapies.

#### Hypotheses.

Firstly, we expect the therapeutic outcomes (symptoms, quality of life and functioning) of the EG_1_ to be significantly better than those obtained in the WG. We also expect the therapeutic outcomes of EG_1_ to be significantly better than those of the EG_2_ group. Furthermore, we expect that the improvements obtained in EG_1_ and EG_2_, respectively, will be maintained at 3- and 9-month follow-up.

Second, we expect therapeutic outcomes to be better in groups led by highly experienced therapists than in groups led by less experienced therapists. In addition, highly experienced therapists will have a better therapeutic alliance, better retention rates and higher attendance rates than groups led by less experienced therapists. Finally, therapeutic outcomes are also expected to be better in groups led by therapists who achieve a higher therapeutic alliance versus those led by therapists with a lower therapeutic alliance.

Finally, patients who use psychotropics for ≥ 3 months are expected to have less symptom reduction than patients who do not use psychotropics or who have taken psychotropics for <three months. Also, patients taking psychotropics for ≥ 3 months are expected to have more neuropsychological disturbances and worse ER than patients who do not use psychotropics or who have taken psychotropics for < 3 months.

## Methods

### Study design

**Fig 1:**
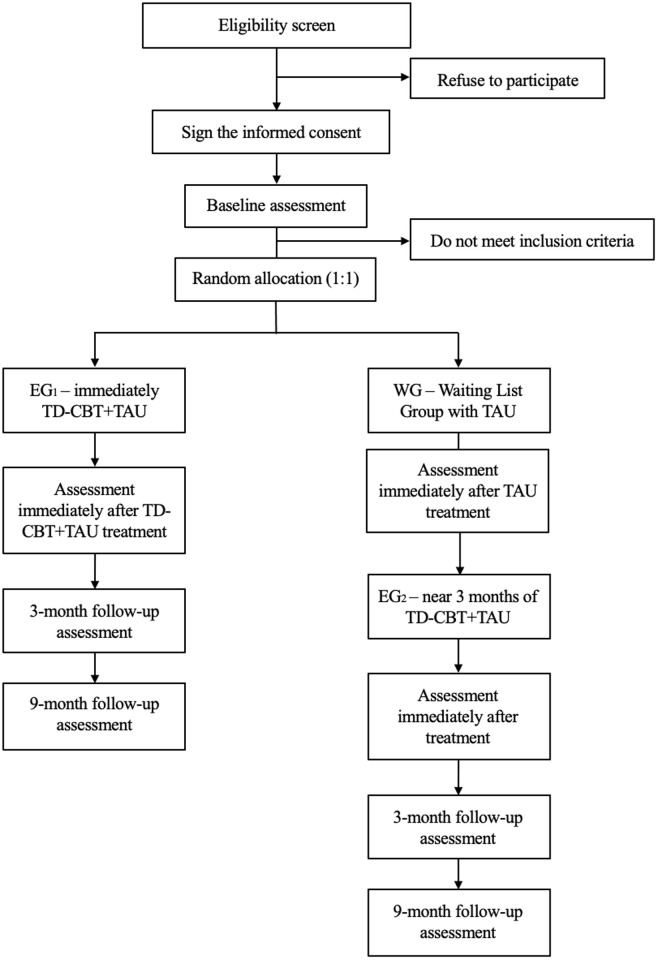
Design Flowchart. EG_1_: experimental group 1; EG_2_: experimental group 2; WG: Waiting Group; TAU: treatment as usual; TD-CBT: transdiagnostic cognitive‐behavioural therapy.

A single blind, multicentre RCT will be carried out at 6 PC centres in the Valencian region of Spain (Alicante and Valencia), following the same method described in the original PsicAP trial [81], but modified to perform an effectiveness trial. In this stepped wedge design, participants who meet the inclusion criteria will be assigned in one “Treatment Package” and blindly randomized to one of two groups: 1) an experimental group (EG_1_; TD-CBT+TAU) or 2) a waiting list group (WG; TAU alone). The treatment (EG_1_) will consist of seven sessions of TD-CBT delivered once per week over a three-month period. Upon completion of the treatment program in EG_1_, all pretreatment variables will be reassessed and both groups (EG_1_ vs. WG) will be compared. Afterwards, patients in the WG will be allocated to receive the same treatment (TD-CBT+TAU), thus forming a second experimental group (EG_2_). This group will receive the same treatment as EG_1_, but delivered approximately three months later. Follow-up assessments will be performed in both experimental groups (EG_1_ and EG_2_) at 3 and 9 months after the posttreatment assessment. Data from these assessments will be used for the within-group analyses. The final 9-month follow-up assessment will be performed approximately one year after the pretreatment assessment (Figure 1).

Each Treatment Package will include several treatments and conditions:

(i) *Treatment as usual* (TAU): medical advice and/or psychotropic medication.
(ii) *Psychological treatment + TAU*: TD-CBT+TAU

Therefore, each will have three different conditions:

(i) *Waiting list group* (WG): TAU
(ii) *Experimental group 1* (EG_1_): TD-CBT+TAU
(iii) *Experimental group 2* (EG_2_): the WG will be converted to an experimental group, which will receive the same treatment as EG_1_ (TD-CBT+TAU), but delivered three months later.

The study was approved by the National Scientific Research Ethics Committee in Spain, conducted in accordance with the Declaration of Helsinki (EUDRACT: 2013-001955-11) and the study protocol was registered (ISRCTN58437086). All participants gave their written informed consent.

### Participants and sample selection

To determine the sample size needed for our stepped wedge trial we used the swCRTdesign package in R. Assuming a dropout rate of 20%, the study will include at least 320 patients, equally distributed in the EG_1_ and WG groups (160 per group). With this sample size, the result will be statistically significant when making comparisons between groups, even if they differ by one point only on the subscales of the PHQ measures, with a standard deviation of 5 (83% statistical power). This will allow us to conclude that the outcome is different for each group at a 95% confidence level. As this was an RCT conducted in a medical-healthcare setting, an intracluster correlation (ICC) of.01 was assumed in the calculations [82].

**Fig 2:**
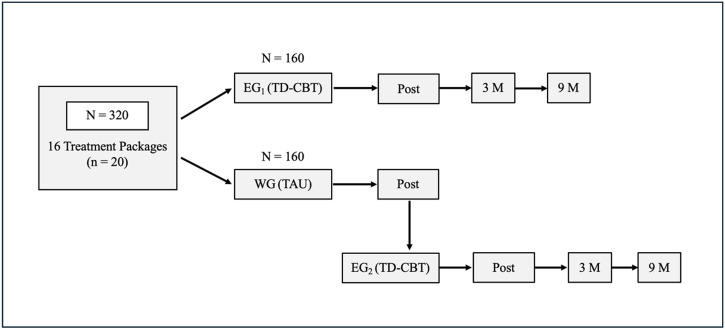
Flowchart of treatment packages. EG_1_: Experimental Group 1; EG_2_: Experimental Group 2; TD-CBT group is plus TAU; TD-CBT: Transdiagnostic-Cognitive Behavioral Therapy; TAU: Treatment as Usual; WG: Waiting Group.

For the first overall objective, we aim to include 320 patients, 160 treated by TD-CBT+TAU in EG_1_ and 160 treated with TAU alone in the WG. Patients will be randomly assigned to EG_1_ or WG in a 1:1 ratio. Assuming 16 treatment packages with 20 patients each (10 assigned to EG_1_ and 10 to WG), 160 patients will be included in each group. All WG groups will be assigned to receive TD-CBT+TAU (EG_2_) approximately 3 months after the start of treatment in EG_1_. At the end, a total of 320 patients will receive TD-CBT+TAU (EG_1_ + EG_2_) (fig 2).

**Fig 3:**
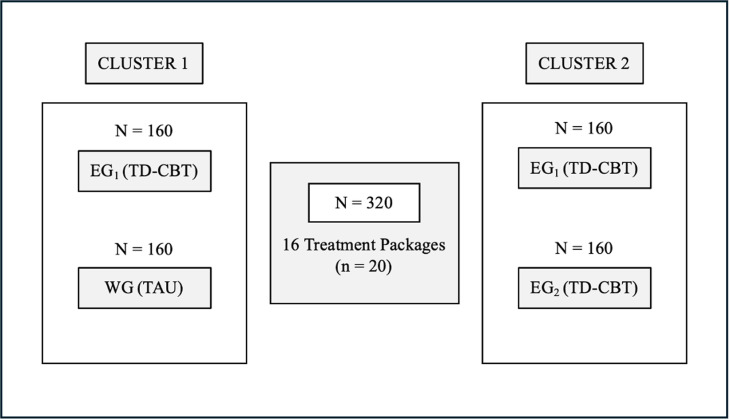
Description of clusters. EG_1_: Experimental Group 1; EG_2_: Experimental Group 2; TD-CBT group is plus TAU; TD-CBT: Transdiagnostic-Cognitive Behavioral Therapy; TAU: Treatment as Usual; WG: Waiting Group.

These 320 patients will be included in two different clusters, as shown in fig 3. Cluster 1 will allow us to compare the EG_1_ and WG groups to determine the most effective treatment approach (i.e., TAU alone or TD-CBT+TAU). Cluster 2 will allow us to compare the two experimental groups, EG_1_ vs. EG_2_, to determine whether psychological treatment provides the same therapeutic benefits 3 months after being on the waiting list.

For the second overall objective, we will select 90 patients from the total sample included in the trial using a computer-generated random sequence. This subsample of 90 patients will be divided into three different groups: no psychotropic medication, short-term use (< 3 months) and long-term use (≥ 3 months). From all these patients, both prescription and use of psychotropic drugs will be collected, in order to study possible discrepancies in the results. It is expected that each group can have about 30 patients and be relatively equal. The 90 patients will undergo an additional assessment by a research psychologist with specific training in neuropsychology that will last approximately 1 hour. During this session, the patient will complete the neuropsychological test battery specified in Table 1. This subsample of patients will be assessed before the start of TD-CBT (if they belong to the WG they will be assessed 3 months after the initial screening session, as this is the established waiting time before becoming EG_2_) and at 12 months.

**Table 1:** Overview of the Assessment Measures. BMQ: Beliefs about Medicines Questionnaire; CERQ-27: Cognitive emotion regulation questionnaire-27; COWAT: Verbal Fluency Test; GAD-7: Generalized Anxiety Disorder-7; GSRS: Group Session Rating Scale; IACTA: Inventory of Cognitive Activity in Anxiety Disorders; MCQ-NB: Metacognitions Questionnaire-Negative Beliefs subscale; PHQ-15: Patient Health Questionnaire-15; PHQ-9: Patient Health Questionnaire-9; PHQ-PD: Patient Health Questionnaire-Panic Disorder; PSWQ-A: Penn State Worry Questionnaire-Abbreviated; ROFC: Rey-Osterrieth Complex Figure Test; RRS-B: Ruminative Responses Scale-Brooding subscale; SPWB: Psychological Wellbeing Scale; SDS: Sheehan Disability Scale; TAVEC: Spain-Complutense Verbal Learning Test; TD-CBT: transdiagnostic cognitive-behavioural therapy; TMT: Trail Making Test; WAI-P: Working Alliance Inventory Patient Form; WAIS-IV: Letters and Numbers subtest; WHOQOL-BREF: WHO Quality of Life scale-Brief version.

Domain	Measure	Reference
Demographics	Age, gender, marital status, education level, family incomes	NA
*Primary outcomes (anxiety and depression)*		
Anxiety Symptoms	Generalised Anxiety Disorder Assessment (GAD-7)	[83]
Depressive symptoms	Patient Health Questionnaire-9 (PHQ-9)	[84]
*Secondary outcomes (somatizations and panic)*		
Somatic symptoms	Patient Health Questionnaire-15 (PHQ-15)	[87]
Panic disorder	Patient Health Questionnaire-Panic Disorder (PHQ-PD)	[88]
*Functioning and Quality of Life*		
Work, family and social functioning	Sheehan Disability Scale (SDS)	[89]
Quality of Life	World Health Organization Quality of life Instrument-Abbreviated version (WhoQoL-Bref)	[90]
Health related-quality of Life	EuroQoL (EQ-5D-5L)	[91]
Psychological Well-Being	Scales of Psychological Well-Being (SPWB)	[92]
*Emotion Regulation Strategies*		
Worry	Penn State Worry Questionnaire – Abbreviated (PSWQ-A)	[93]
Rumination	Ruminative Responses Scale – Brooding (RRS-B)	[94]
Negative metacognitions	The Metacognitions Questionnaire–Negative Beliefs (MCQ-NB)	[95]
Attentional biases	Inventory of Cognitive Activity – Panic Brief (IACTA-PB)	[96]
Cognitive emotion regulation	Cognitive Emotion Regulation Questionnaire (CERQ-27)	[97]
*Therapeutic effectiveness*		
Patient Alliance with the therapist	Working Alliance Inventory – Patient Form (WAI-P)	[98]
Patient Alliance with the group of patients	Group Session Rating Scale (GSRS)	[99]
Therapist experience	Ad-hoc questionnaire: years of experience, training level and gender	NA
Number of sessions	Ad-hoc questionnaire: Number of sessions attended	NA
Treatment satisfaction	Ad-hoc question: treatment satisfaction (1–10)	NA
*Neuropsychological measures*		
Visual attention and processing speed	Trail Making Test (TMT)	[100,101]
Visual flexibility and cognitive inhibition	Stroop Test	[102,103]
Visual perception, construction and memory	Rey-Osterrieth Complex Figure Test (ROFC)	[104]
Visual processing speed	Symbol Digit Modalities Test (SDMT)	[105]
Remote and working memory	Digits Subtest of the WAIS-IV	[106]
Working memory	Letters and Numbers Subtest of the WAIS-IV	[106]
Verbal memory and learning	Spain-Complutense Verbal Learning Test (TAVEC)	[107]
Verbal Fluency	Controlled Oral Word Association Test (COWAT)	[108]
*Psychotropic medication*		
Prescription of medication	Ad-hoc questionnaire: Prescription, type, dose and time of use	NA
Medication use	Ad-hoc questionnaire: Current use, type, dose and time of use	NA
Beliefs about psychotropic medication	Beliefs about medicines questionnaire (BMQ)	[109]

### Participant recruitment

Patient recruitment will be performed by general practitioners (GPs) at the six participating PC centres. Participating GPs who identify a patient with a suspected diagnosis of an ED will invite the patient to participate in the trial. The patient will be given an information sheet describing the trial and asked to sign an informed consent form. Once the consent form has been signed, a psychologist will contact the patient to schedule an initial appointment during which the psychologist will 1) ensure that the patient meets the inclusion criteria and 2) administer the study instruments (see below for more detailed information).

Patients included who meet the criteria of any of the three groups of pharmacological treatment will be invited to participate in the neuropsychological assessment until a total of 90 patients (30 per group) have been recruited. Once the patients will be randomly assigned to the psychological therapy (EG), and before initiating the sessions, the neuropsychological assessment will then be scheduled and, at this time, the participant’s psychotropic use will be recorded.

Given the characteristics of the trial, patient recruitment dates to complete a post-treatment sample of 200 patients are expected to be between 01/05/2023 and 01/12/2024. The approximate end date of the trial, including 3- and 9-month follow-ups of the last patients recruited, is estimated to be around 01/10/2025.

### Inclusion and exclusion criteria

The study sample will comprise patients with mild, moderate, or moderate-severe symptoms of anxiety and/or depression. For inclusion, patients must meet the pre-determined cut-off score on at least one of the two study scales: GAD-7 (≥10) or PHQ-9 (≥10)[83,84].

Exclusion criteria are as follows: severe mental disorder (e.g., bipolar or personality disorder); substance use disorder; recent suicide attempt; severe disability according to the Sheehan Disability Scale (≥ 25 total points on the first three items); and severe mood disorder (≥ 23 points on the PHQ-9 depression subscale). Note that patients who score 20–23 points on the PHQ-9 depression subscale may be eligible for inclusion pending a clinical interview to rule out severe depression [83]. Patients who do not reach the minimum cut-off points on the GAD-7 or PHQ-9 and those who do not meet criteria for a probable ED will be excluded. Patients who are excluded from the study but suspected to have a severe mental disorder will be referred to their GP for further evaluation and treatment, including a possible referral to specialized care.

### Interventions

#### Transdiagnostic psychological intervention.

The experimental group will receive a total of seven sessions (1.5 h/session) of TD-CBT delivered in small groups (8–10 persons). The programme will be delivered over a period of 10–12 weeks (approximately 3 months). Initially, sessions will be scheduled weekly, however, over time, the interval between sessions will be progressively increased (to see more information [85]). After the seven regular sessions of the original PsicAP protocol, those patients who do not obtain a reliable recovery in the post-treatment assessment will be eligible to receive up to three individual therapeutic sessions in the following three months (one session per month). These sessions will work on individual aspects of each patient’s situation using the transdiagnostic tools provided during the group protocol. It is hoped that in this way the person will learn to apply these tools more specifically and achieve reliable recovery.

#### Treatment-as-usual.

The TAU group will receive the standard treatment prescribed by their GP in routine clinical practice [86]. The specific treatment will depend on the individual GP (usually either pharmacological treatment or observation).

### Therapist and training

Three types of therapists will participate in this trial, all of them university graduates: Psychologists Specializing in Clinical Psychology (PSCP), General Health Psychologists (GHP) and General Health Psychologists recently graduated from the qualifying master’s degree (GHPM). To work as a PSCP in the Spanish National Health System (NHS), it is necessary to complete an Internal Residency Programme (IRP) in the NHS, which consists of 4 years of work and training under the supervision of a specialist. The PSCPs in this trial have more than 30 years of experience. GHP are psychologists qualified to work in the health care setting, who in our trial have at least 10 years of clinical experience. Finally, GHPMs are GHPs who are recent graduates of the qualifying master’s degree and who have 1 year of clinical experience. As can be seen, there is a hierarchy in the level of training and experience among therapists, with PSCPs being the most competent psychologists. Throughout the trial, GHPs, and especially GHPMs, will be supervised by PSCPs in regular clinical sessions.

In addition to regular training, all therapists will undergo a standardized training programme on the PsicAP protocol, conducted by a supervisor and trainer. This training consists of studying the Therapist Manual, four internet-based lessons on the content of each session and a face-to-face session with the trainer. This course must be completed before any psychologist can provide group therapy as part of the trial.

Given that the drop-out rate in this type of trial can be high (especially in medium and long-term follow-up), two strategies will be implemented. On the one hand, during the psychological treatment, during sessions 2 and 4, a small amount of time will be dedicated to reminding patients of the importance of adherence to therapy and homework tasks. On the other hand, after the post-treatment assessment, a psychologist from the project will make phone calls to patients every six weeks (approximately 10–15 minutes). The purpose of these calls is to reinforce the therapeutic strategies learned during the group sessions and to monitor the emotional well-being of the participants. It is hoped that these strategies will help reduce drop-out rates.

### Outcomes

An overview of the assessment measures is provided in Table 1 [83,84,87–109]. The sequence of data collection is shown in Table 2.

**Table 2:** Study timeline according to SPIRIT Statement. BMQ: Beliefs about Medicines Questionnaire; CERQ-27: Cognitive emotion regulation questionnaire-27; COWAT: Verbal Fluency Test; GAD-7: Generalized Anxiety Disorder-7; GSRS: Group Session Rating Scale; IACTA: Inventory of Cognitive Activity in Anxiety Disorders; MCQ-NB: Metacognitions Questionnaire-Negative Beliefs subscale; PHQ-15: Patient Health Questionnaire-15; PHQ-9: Patient Health Questionnaire-9; PHQ-PD: Patient Health Questionnaire-Panic Disorder; PSWQ-A: Penn State Worry Questionnaire-Abbreviated; ROFC: Rey-Osterrieth Complex Figure Test; RRS-B: Ruminative Responses Scale-Brooding subscale; SPWB: Psychological Wellbeing Scale; SDS: Sheehan Disability Scale; TAVEC: Spain-Complutense Verbal Learning Test; TD-CBT: transdiagnostic cognitive-behavioural therapy; TMT: Trail Making Test; WAI-P: Working Alliance Inventory Patient Form; WAIS-IV: Letters and Numbers subtest; WHOQOL-BREF: WHO Quality of Life scale-Brief version.

	PRINCIPAL STUDY PERIOD											
	**Recruitment**	**Allocation**	**Intervention (no. sessions)**							**Post-treatment**		
**TIMEPOINT**	*-t*_*1*_	**0**	*1*	*2*	*3*	*4*	*5*	*6*	*7*	**Immediately after treatment**	**3 months**	**9 months**
**ENROLMENT:**												
GP Invitation	**×**											
Informed consent	**×**											
Allocation		**×**										
**INTERVENTIONS:**												
Group TD-CBT (EG_1_ & EG_2_)		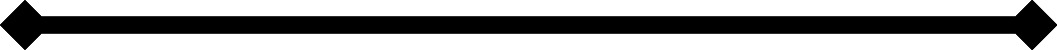										
Waiting List Group TD-CBT		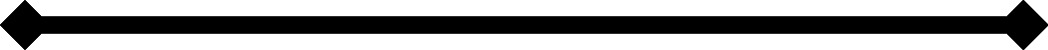										
**ASSESSMENTS:**												
**Symptom measures (Primary outcomes)**												
PHQ-9	**×**	**×**		**×**		**×**				**×**	**×**	**×**
GAD-7	**×**	**×**		**×**		**×**				**×**	**×**	**×**
**Symptom measures (Secondary outcomes)**												
PHQ-15	**×**			**×**		**×**				**×**	**×**	**×**
PHQ-PD	**×**			**×**		**×**				**×**	**×**	**×**
**Measures of Functioning and Quality of Life**												
SDS	**×**	**×**								**×**	**×**	**×**
WhoQoL-Bref	**×**									**×**	**×**	**×**
EQ-5D-5L	**×**									**×**	**×**	**×**
SPWB	**×**									**×**	**×**	**×**
**Measures of Emotion Regulation Strategies**												
PSWQ-A	**×**									**×**	**×**	**×**
RRS-B	**×**									**×**	**×**	**×**
MCQ-NB	**×**									**×**	**×**	**×**
IACTA-PB	**×**									**×**	**×**	**×**
CERQ-27	**×**									**×**	**×**	**×**
**Therapeutic effectiveness**										**×**		
WAI-P				**×**		**×**				**×**		
GSRS				**×**		**×**				**×**		
Therapist Experience	**×**									**×**	**×**	**×**
Number of sessions										**×**	**×**	
Treatment Satisfaction										**×**	**×**	
**Neuropsychological Measures**												
TMT	**×**											**×**
Stroop Test	**×**											**×**
ROFC	**×**											**×**
SDMT	**×**											**×**
WAIS-IV (D; L&N)	**×**											**×**
TAVEC	**×**											**×**
COWAT	**×**											**×**
**Measures of psychotropics**												
Ad hoc questionnaire	**×**									**×**	**×**	**×**
BMQ	**×**											**×**

### Statistical analysis

Data will be analysed and coded using SPSS (v.29.0), MPLUS and R. To assess treatment efficacy, primary analyses will be conducted on an intention-to-treat (ITT) basis and compared with secondary analyses on a per-protocol (PP) basis. The ITT-based approach preserves randomization of participants and includes all initially included patients. This approach has proven to be more robust than PP approaches in RCTs, especially when wanting to assess the pragmatism and implementation of a treatment in real-world settings [110], one of the main objectives of this stepped wedge RCT.

General Linear Mixed Models (GLMM) will be used for the analysis of treatment effectiveness, as these models have been shown to be particularly useful when working with data that have a hierarchical or cluster structure, such as longitudinal or repeated measures data [111,112]. In all cases, time (pre-treatment; post-treatment; 3 and 9 months) and group (EG_1_ or WG/EG_2_) will be included as fixed effects, and subjects, treatment package and therapist as random effects. The main interactions of each model will also be studied. In addition, pre-treatment scores for each target variable, age, level of incomes, gender and educational attainment will be introduced as covariates. In all models, fixed, random and covariate effects will be held constant and only the target dependent variable will be modified: anxiety, depression, QoL, functioning or ERS. For the analysis of neuropsychological function (attention, memory, learning, etc.) we will proceed in a similar way, but we will include the variable time of taking drugs (not taking < 3 months, ≥ 3 months) as another fixed effect to be studied. In all analyses an α level ≤.05 will be set and effect sizes will be calculated using the Morris d, which takes into account the mean and standard deviation of the sample at both the final assessment and at baseline, resulting in a more representative effect size [113].

Finally, recovery, reliable recovery and deterioration rates will also be calculated. These analyses only take into account anxiety (GAD-7) and depression (PHQ-9) scores. Recovery rate can be defined as pre-treatment scores at or above the inclusion threshold on either scale (≥10) and below the threshold on both scales at post-treatment assessment (<10). The reliable recovery rate will be calculated using a change score based on the standard deviation and Cronbach’s alpha of each measure to account for measurement errors of the scales [114]. Thus, a change score ≥ 5 will be used for the GAD-7 and ≥ 6 for the PHQ-9. Thus, those patients who achieve recovery, and furthermore do so with these change scores on at least one of the two subscales, will be categorized as reliable recovery. Finally, deterioration will be defined as an increase in score on either scale compared to pre-treatment, while meeting the change score criteria described for reliable recovery on at least one of the two scales.

## Discussion

The results of this trial are expected to improve our understanding of evidence-based transdiagnostic psychological treatment in the PC setting. The PsicAP treatment protocol has been proven to reduce symptoms and improve functioning and QoL in patients with different EDs [17]. Studies carried out to investigated psychological processes related to transdiagnostic treatment [41,115] have identified several mechanisms of change and ERS are associated with treatment outcomes. The next step is to evaluate whether other mechanisms of change, including other adaptive ERS, therapist experience, and therapeutic alliance, play a role in improving treatment adherence and outcomes. This trial will also assess the impact of antidepressant and/or anxiolytic use on neuropsychological function and how this influences treatment effectiveness.

We expect this effectiveness trial to provide novel insights into the practicality of implementing the PsicAP protocol in PC settings in the real-world. We also hope to study the differential impact of different degrees of therapist experience and therapeutic alliance to treatment on programme outcomes. Previous studies have shown that a lack of therapeutic alliance (or within-group alliance) is an important barrier to success in psychological therapy in PC, especially in patients with symptoms of anxiety and depression [50]. In this regard, it may be possible to improve treatment adherence by obtaining a better understanding of the influence of therapeutic processes such as therapist experience and therapeutic alliance. In fact, an important aim of this project is to examine how certain factors influence treatment adherence. For example, a recent study based on IAPT data [116] found that patients who self-referred were more likely to attend their appointment. That study also found that other factors—older age, fewer previous referrals, and consenting to receive reminder messages—also increased the probability of attending the sessions. Also, there is an association between psychotropic use and adherence. A meta-analysis by Swift et al. [49] found that patients on pharmacotherapy had a higher dropout rate than patients receiving psychotherapy alone. In that study, patients with depressive disorders on pharmacotherapy were 1.26 times more likely to drop out than those receiving psychological treatment.

A novel aspect of this clinical trial is the study of the impact of psychotropics (antidepressants and anxiolytics), particularly long-term use, on neuropsychological function and how these changes influence treatment outcomes. It is crucial to understand this relationship, particularly given the growing use of psychotropics in many countries, including Spain, where 8,6% of adults are taking antidepressants. Despite the widespread use of antidepressants, long-term safety and effectiveness data are scant, mainly because most studies performed to date (many with important methodological limitations) have only evaluated the short-term effects (6–8 weeks) [57,60]. In this trial, we expect to present long-term follow-up data to better elucidate the long-term neuropsychological effects of psychotropic drugs in patients with ED. This trial will help also to provide data on the effects of both short and long-term use of psychotropics on patients’ mental health and well-being, in terms of emotional symptoms, QoL, therapeutic effectiveness, and neuropsychological function. This will show how common medications used to treat depression and anxiety affect patients’ ability to participate in and benefit from psychological therapy. Preliminary data from some studies show that the prescription of psychotropics may negatively affect therapeutic outcomes [117,118]. This trial should help clarify the effects of these medications, both on cognitive function and treatment outcomes, and could help to determine whether minimizing the duration of exposure to these medications could improve well-being. If true, this would improve treatment effectiveness and QoL.

This RCT has several potential limitations. First, although the stepped wedge design allows for masked randomization, treatment outcomes can only be assessed at the posttreatment assessment, as the WG will be switched to an experimental group (TD-CBT) after that analysis. This means that follow-up at 3 and 9 months will be only comparable for within-groups analyses, which could increase the risk of bias. However, assuming that the posttreatment and follow-up results are similar to those obtained in the original PsicAP trial, we could infer that the long-term effects are explained by adding TD-CBT to TAU. There is also another component that may interfere with the results of the WG group, the expectation of receiving psychological therapy. In this sense, it is possible that patients randomized to the WG group improve their scores more before starting group therapy than a strict control group where the patient knows that he/she will not receive psychological treatment. However, this may also be a window of opportunity to compare the results with the original PsicAP data, as this could be an indication for future studies on the psychological effect of therapeutic hope. Likewise, in stepped wedge designs, there is also the possibility of a higher experimental mortality rate in EG2, as some WG patients may change their initial situation due to the effect of time and drop out or be excluded from the RCT before starting the experimental treatment. This is in addition to the fact that the drop-out rate in this type of RCTs conducted in PC is usually high [119]. To mitigate the effects of this limitation, an ITT data analysis approach will be used, which as discussed above is suitable for this type of RCT and helps to reduce potential biases related to experimental dropout.

Another important limitation is the possible difference in therapeutic alliance and adherence generated by different types of therapists, which may lead to a higher dropout rate or worse therapeutic outcomes. However, although this may be seen as a limitation, it is also one of the main study objectives of this RCT and may provide important information on the feasibility and safety of including therapists with different levels of training and experience in the PC setting. Finally, there are some limitations related to the accuracy of the data on psychotropic drug use and the neuropsychology sub-study. Firstly, there may be a difference between the prescriptions made by the general practitioner and the actual consumption by the patient. Secondly, it is possible that the sub-sample of neuropsychologically assessed patients may present a high level of heterogeneity in terms of the number of patients per subgroup of psychotropic drug use. To mitigate these limitations, we will collect both prescription and actual consumption data. We will also record the type of drug (antidepressant, anxiolytic or hypnotic); the time of use (expressed in months); and the daily dose.

The results of this trial could have several important clinical implications. First, it will demonstrate the potential impact of short- and long-term psychotropic use on cognitive function, emotional symptoms, functioning, and QoL. If, as we suspect, our results show that these drugs have a negative impact on treatment outcomes, this could provide further support for the greater use of evidence-based non-pharmacological and transdiagnostic strategies [16,17]. This is of particular relevance since, as noted in the introduction, a significant number of current patients develop a high level of dependence on psychotropic drugs, as well as a strong withdrawal syndrome at the time of treatment discontinuation [77]. Our findings could help to reduce daily overmedicalization of mild and moderate psychological problems and introduce more effective, potentially less harmful and increasingly preferred solutions for patients [120]. Furthermore, as other studies internationally have already pointed out, reducing the use of purely pharmacological strategies for the treatment of EDs in the PC setting can save large amounts of money for the NHS [18,121]. Second, the use of a stepped wedge design, together with the individualized therapy option in patients who fail to achieve a reliable recovery (even after completing TD-CBT), may provide support for the value and feasibility of implementing stepped care model for mental health treatment, similar to the offered the IAPT in the UK. In this stepped-care model, patients with mild or moderate disorders would receive the PsicAP protocol (TD-CBT in group format), while those with more severe ED would receive a “boost” in the form of up to three individual sessions. This stepped care model [122] could also help to reduce over-medication and thus reduce costs to the healthcare system [123].

In conclusion, we expect that this effectiveness trial will help to better understand how to implement an evidence-based psychological treatment protocol in the Spanish PC setting in order to improve treatment outcomes versus TAU while simultaneously reducing the costs (both direct and indirect) associated with EDs, both in the short and long term.

## Abbreviations

BMQ: Beliefs about Medicine Questionnaire
CERQ-27: Cognitive emotion regulation questionnaire-27
COWA: Verbal Fluency Test
ED: Emotional Disorder
EG: Experimental Group
EQ-5D-5L: EuroQol
ER: Emotion Regulation
ERS: Emotion Regulation Strategies
GAD-7: Generalized Anxiety Disorder-7
GHP: General Health Psychologists
GHPM: General Health Psychologists recently graduated from the qualifying master’s degree
GP: General Practitioner
GSRS: Group Session Rating Scale
IACTA: Inventory of Cognitive Activity in Anxiety Disorders
IAPT: Improving Access to Psychological Therapies
MCQ-NB: Metacognitions Questionnaire-Negative Beliefs subscale
NHS: National Health System
PC: Primary Care
PCSP: Psychologists Specializing in Clinical Psychology
PD: Panic Disorder
PHQ-15: Patient Health Questionnaire-15
PHQ-9: Patient Health Questionnaire-9
PHQ-PD: Patient Health Questionnaire-Panic Disorder
PSWQ-A: Penn State Worry Questionnaire-Abbreviated
QoL: Quality of Life
RCT: Randomized Controlled Trial
ROFC: Rey-Osterrieth Complex Figure Test
SPWB: Psychological Wellbeing Scale
RRS-B: Ruminative Responses Scale-Brooding subscale
SDS: Sheehan Disability Scale
TAU: Treatment As Usual
TAVEC: Spain-Complutense Verbal Learning Test
TD-CBT: transdiagnostic cognitive-behavioral therapy
TMT: Trail Making Test
WAI-P: Working Alliance Inventory Patient Form
WAIS-IV: Letters and Numbers subtest
WG: Waiting Group
WHOQOL-BREF: WHO Quality of Life scale-Brief version
